# Endoscopic closure using an over‐the‐scope clip for pancreatobiliary endoscopy‐related large gastrointestinal perforation (with video)

**DOI:** 10.1002/deo2.48

**Published:** 2021-08-22

**Authors:** Akashi Fujita, Tomoaki Tashima, Yuki Tanisaka, Masafumi Mizuide, Tomoya Ogawa, Yoichi Saito, Hiromune Katsuda, Kazuya Miyaguchi, Yumi Mashimo, Yuya Nakano, Rie Terada, Ryuhei Jinushi, Shomei Ryozawa

**Affiliations:** ^1^ Department of Gastroenterology Saitama Medical University International Medical Center Hidaka Saitama Japan

**Keywords:** endoscopic retrograde cholangiopancreatography, endoscopic ultrasound, gastrointestinal perforation, over‐the‐scope clip

## Abstract

Endoscopic ultrasonography (EUS) and endoscopic retrograde cholangiopancreatography (ERCP) for pancreatobiliary diseases require advanced techniques. We usually use an oblique‐viewing endoscope in such procedures. Sometimes, tumor invasion causes gastrointestinal strictures. Crossing a stricture using an oblique‐viewing endoscope is more difficult than using a forward‐viewing scope. Therefore, the frequency of scope perforation is higher than other endoscopic procedures. Although surgical repair for gastrointestinal perforations caused by endoscopes has been performed, patients with pancreatobiliary diseases are often elderly and in poor general condition; therefore, patients are hesitant to undergo surgical treatments. Recently, the usefulness of over‐the‐scope clipping (OTSC) as a minimally invasive rescue method has also been reported. In this study, we report cases of successful endoscopic closure using OTSC for gastrointestinal perforations caused by endoscopes in ERCP and EUS‐related procedures. After those procedures, all cases showed no abnormalities in blood tests or symptoms, and emergency surgery was successfully avoided. Thus, endoscopic closure using OTSC for pancreatobiliary endoscopy‐related gastrointestinal perforations is safe and effective. However, OTSC requires some expertise. A good assessment of defect size and careful insertion of the scope using OTSC attached to the upper esophagus are needed to avoid clip migration or disinsertion and esophageal tears. Therefore, endoscopic closure using OTSC could be the first choice of treatment for pancreatobiliary endoscopy‐related gastrointestinal perforations. We should be familiar with its indication and perform it carefully and rapidly.

## INTRODUCTION

Endoscopic ultrasonography (EUS) and endoscopic retrograde cholangiopancreatography (ERCP) for pancreatobiliary diseases require advanced techniques. Usually, an oblique‐viewing endoscope is used in such procedures. Sometimes, tumor invasion causes gastrointestinal strictures. Crossing a stricture using an oblique‐viewing endoscope is more difficult than using a forward‐viewing scope. Therefore, the frequency of complications is higher than other endoscopic procedures, and the incidence of scope‐induced perforations has been reported to be 0.02%–1.8%.^1^, ^2^, ^3^ Although surgical repair for endoscope‐induced gastrointestinal perforations has been performed, patients with pancreatobiliary diseases are often elderly and in poor general condition and, thus, are hesitant to undergo surgery. Recently, the usefulness of an over‐the‐scope clip (OTSC) as a minimally invasive rescue method has also been reported.^4^ In this study, we report cases of successful endoscopic closure using OTSC for endoscope‐induced gastrointestinal perforations in ERCP and EUS‐related procedures.

## CASE REPORT

Between August 2017 and May 2021, we performed endoscopic closure using OTSC for pancreatobiliary endoscopy‐related gastrointestinal perforation in four cases. A convex linear‐array echoendoscope (GF‐UCT260; Olympus, Tokyo, Japan) was used for the EUS‐related procedures, and an oblique‐viewing duodenoscope (TJF260V; Olympus, Tokyo, Japan) was used for ERCP‐related procedures. These cases complied with the Declaration of Helsinki, as revised in Brazil in 2013. All patients provided a written informed consent for EUS‐ and ERCP‐related procedures. A summary of these cases is shown in Table 1.

**TABLE 1:** Summary of the four cases

Case	Defect size	Defect location	Procedure time*	Primary disease	Response to primary disease
1	13 mm	SDA	15 min	PDAC	Surgery
2	12 mm	SDA	14 min	PDAC	Chemotherapy
3	12 mm	EG junction	18 min	pNEN	Surgery
4	20 mm	D2	22 min	BDCA	BSC

Abbreviations: BDCA, bile duct cancer; BSC, best supportive care; D2, second part of the duodenum; EG junction, esophagogastric junction; PDAC, pancreatic ductal adenocarcinoma; pNEN, pancreatic neuroendocrine neoplasm; SDA, superior duodenal angle.

* Time between the diagnosis and the application of an over‐the‐scope clip.

### OTSC procedure

**FIGURE 1:**
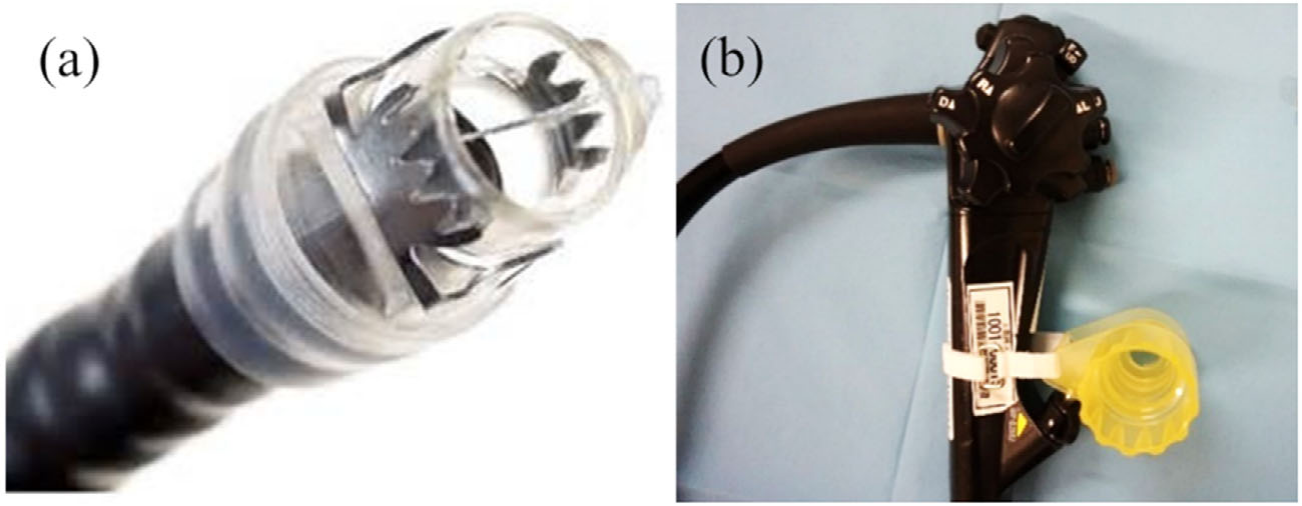
The over‐the‐scope clip system. (a) A clip with an applicator cap. (b) A hand wheel to deploy the clip

A 9‐mm OTSC (Ovesco Endoscopy GmbH, Tuebingen) (Figure 1) and forward‐viewing endoscope (GIF‐Q260J or GIF‐H290T; Olympus, Tokyo, Japan) were used for endoscopic closure. After recognizing the perforation, the ERCP and EUS‐related procedures were immediately discontinued, and a 9‐mm OTSC was attached to the tip of the forward‐viewing scope. We carefully reinserted the scope and confirmed the perforation site. Subsequently, we sufficiently suctioned the lesion into the attachment cap and placed the OTSC (Video S1).

### Case 1

A woman in her 70s with pancreatic head tumor was referred to our hospital.

**FIGURE 2:**
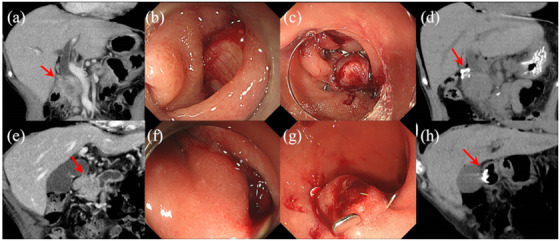
Case 1 (upper figure) and case 2 (lower figure). (a) Pancreatic head tumor on computed tomography (CT) (red arrow). (b) A large perforation, measuring approximately 13 mm in diameter, occurred in the posterior wall of the superior duodenal angle (SDA). (c) The wound filled with the omentum after placement of an over‐the‐scope clip (OTSC). (d) The OTSC detected on CT (red arrow) after the endoscopic repair. (e) Pancreatic head tumor on CT (red arrow). (f) A large perforation, measuring approximately 12 mm in diameter, occurred in the posterior wall of the SDA. (g) The wound filled with the surrounding tissue after placement of an OTSC. (h) The OTSC detected on CT (red arrow) after the endoscopic repair

We suspected pancreatic head cancer on computed tomography (CT) (Figure 2a) and planned endoscopic ultrasound‐guided fine needle aspiration (EUS‐FNA) to obtain histological evidence before surgery. However, while inserting an echoendoscope into the second part of the duodenum, a large perforation, measuring approximately 13 mm in diameter, occurred in the posterior wall of the superior duodenal angle (SDA) (Figure 2b). After recognizing the perforation, the procedure was immediately discontinued, and OTSC was placed. It was confirmed that the wound had been filled with the omentum (Figure 2c). After the procedure, the patient successfully recovered without developing peritonitis (Figure 2d). EUS‐FNA was not re‐attempted and pancreatic head cancer surgery was performed.

### Case 2

A man in his 80s with pancreatic head tumor was referred to our hospital.

We suspected pancreatic head cancer on CT (Figure 2e) and planned EUS‐FNA to obtain histological evidence before chemotherapy. However, while inserting an echoendoscope into the second part of the duodenum, a large perforation, measuring approximately 12 mm in diameter, occurred in the posterior wall of the SDA (Figure 2f). The procedure was immediately discontinued, and OTSC was placed (Figure 2g). After the procedure, the patient successfully recovered without developing peritonitis (Figure 2h). EUS‐FNA was not re‐attempted, and chemotherapy for pancreatic head cancer was performed.

### Case 3

A man in his 70s with pancreatic head tumor was referred to our hospital.

**FIGURE 3:**
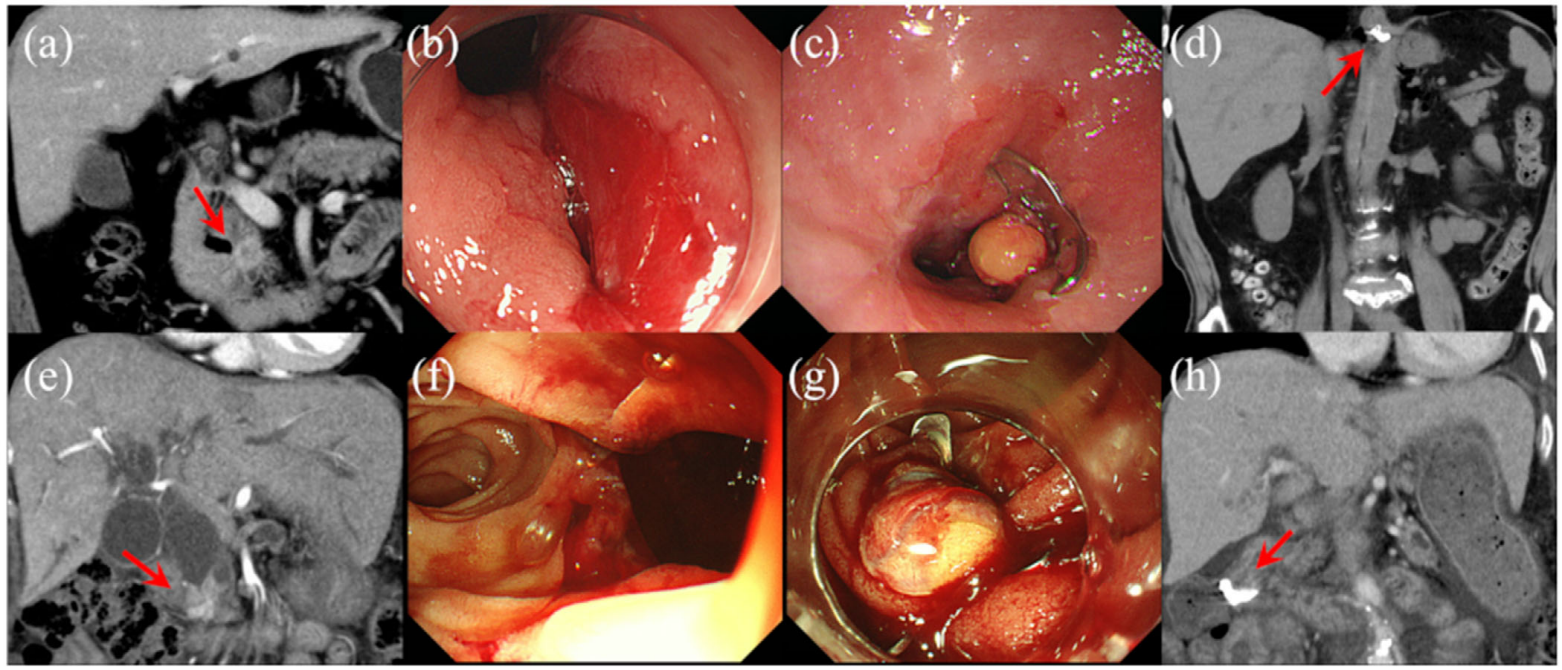
Case 3 (upper figure) and case 4 (lower figure). (a) Pancreatic head tumor on computed tomography (CT) (red arrow). (b) A large perforation, measuring approximately 12 mm in diameter, occurred in the posterior wall of the esophagogastric junction. (c) The wound filled with fatty tissue after placement of an over‐the‐scope clip (OTSC). (d) The OTSC detected on CT (red arrow) after the endoscopic repair. (e) Distal bile duct stenosis on CT (red arrow). (f) A large perforation, measuring approximately 20 mm in diameter, occurred in the posterior wall of the second part of the duodenum. (g) The wound filled with the omentum after placement of an OTSC. (h) The OTSC detected on CT (red arrow) after the endoscopic repair

We suspected pancreatic neuroendocrine neoplasm (pNEN) on CT (Figure 3a) and planned EUS‐FNA to obtain histological evidence before surgery. However, while inserting an echoendoscope into the stomach, a large perforation, measuring approximately 12 mm in diameter, occurred in the posterior wall of the esophagogastric junction (Figure 3b). The procedure was immediately discontinued, and OTSC was placed. It was confirmed that the wound had been filled with fatty tissue (Figure 3c). After the procedure, the patient successfully recovered without developing peritonitis (Figure 3d). EUS‐FNA was not re‐attempted and pNEN surgery was performed.

### Case 4

A woman in her 80s with obstructive jaundice was referred to our hospital.

We suspected distal bile duct cancer on CT (Figure 3e) and planned ERCP for bile duct drainage. During duodenoscope manipulation, a large perforation, measuring approximately 20 mm in diameter, occurred in the posterior wall of the second part of the duodenum (Figure 3f). The procedure was immediately discontinued, and OTSC was placed. We considered that the perforation site could be filled with the omentum (Figure 3g). After the procedure, the patient successfully recovered without developing peritonitis (Figure 3h) and underwent percutaneous trans‐hepatic biliary drainage.

## DISCUSSION

Recently, endoscopic diagnosis and treatment in the pancreatobiliary regions have made remarkable technological progress.^5^ Alternatively, the risk of complications is always present and sometimes severe. A patient's condition may worsen as time goes on from the endoscopic perforation of the gastrointestinal tract. Therefore, rapid and reliable defect closure is necessary. Although emergency surgery has been the mainstay of treatment for endoscopic perforations, it is highly invasive, and some reports have indicated a high postoperative mortality rate.^6^ Therefore, minimally invasive endoscopic closure should be considered if possible.

Previously, metallic hemoclips were often used for endoscopic closure, but they only pull the mucosal and submucosal layers, making it difficult to close full‐thickness defects of the gastrointestinal tract, and are not suitable for treating large perforations. Alternatively, OTSC can close full‐thickness defects up to 30 mm,^7^ although it is a relatively new device, and reported cases are limited. Training is needed to perform this procedure because of its similarity with endoscopic band ligation. OTSC is carefully placed while suctioning both sides of the normal mucosa around the perforated site into the application cap. Sometimes, the wound can be filled with the surrounding omentum or fatty tissue.^4^, ^8^ The long‐term adverse events of this procedure have not been reported till date.

In this case report, endoscopic closure using OTSC was performed because we thought that emergency surgery under general anesthesia should be avoided as much as possible by performing rapid and minimally invasive repair after the perforation. After those procedures, all cases showed no abnormalities in blood tests or symptoms, and emergency surgery was avoided. Surgery was performed for primary disease 10 days and 1 month after the perforation in cases 1 and 3, respectively. Due to the perforation, intraoperatively, the abdominal cavity only had minor adhesions which had little effect on the surgical technique. Therefore, endoscopic closure using an OTSC for pancreatobiliary endoscopy‐related gastrointestinal perforations is safe and effective. However, OTSC procedures require some expertise. A good defect size assessment and careful scope insertion with an OTSC attached through the upper esophagus are warranted to avoid clip migration or disinsertion and esophageal tears. It is not indicated in the vicinity of the papilla of Vater due to pancreatic and bile duct obstruction or in the gastrointestinal stenosis where obstruction may occur by this procedure.^9^

According to the European Society of Gastrointestinal Endoscopy guidelines for managing endoscopic perforations of the gastrointestinal tract,^10^ surgical repair is recommended if the perforation is diagnosed several hours after the endoscopic procedure, and the patient shows peritonitis or intra‐abdominal fluid accumulation. Therefore, it is important to consider indications and perform OTSC procedures carefully while consulting a surgeon. Additionally, flexible approaches other than conservative treatments are necessary if poor progress is noted after the procedure. Limitations of this study are a single‐center study design involving a limited number of patients; therefore, further study is warranted to confirm OTSC usefulness.

In conclusion, endoscopic closure using an OTSC for pancreatobiliary endoscopy‐related gastrointestinal perforations could be the first treatment of choice. We should be familiar with its indication and perform OTSC procedures carefully and rapidly.

## CONFLICT OF INTEREST

The authors declare that there is no conflict of interest that could be perceived as prejudicing the impartiality of the research reported.

## FUNDING INFORMATION

None.

## Supporting information


**Video S1**: Endoscopic closure using an over‐the‐scope clip for pancreatobiliary endoscopy‐related gastrointestinal perforation.
